# The Prevention and Treatment of Postpartum Haemorrhage in Domiciliary Practice
*A paper delivered to the Bristol Medico-Chirurgical Society on 13th February, 1952.


**Published:** 1952-04

**Authors:** Louise McIlroy

**Affiliations:** Consulting Obstetric and Gynaecological Surgeon, Royal Free Hospital


### THE PREVENTION AND TREATMENT OF POSTPARTUM HAEMORRHAGE IN DOMICILIARY PRACTICE*

BY

DAME LOUISE McILROY, M.D., F.R.C.P., F.R.C.O.G.

*Consulting Obstetric and Gynaecological Surgeon, Royal Free Hospital*

Postpartum haemorrhage can be a terrifying emergency and the life of the patient depends upon the promptitude of treatment. A young practitioner may meet it in domiciliary practice either at home or abroad, possibly where hospital accommodation for maternity cases is limited, or where a hospital is a long way away. So he must know how to avoid this haemorrhage by proper management of the third stage of labour and how to treat it when it does occur.

### CAUSES OF POSTPARTUM HAEMORRHAGE AND THEIR PREVENTION

The two main causes of postpartum haemorrhage are atony of the uterus, and trauma of the pelvic tissues.

Atony of the uterus is associated with failure of the muscular fibres to contract and retract and obliterate the thin walled sinuses in the placenta area, blood fails to clot and vessel openings fail to retract. Atony of the uterus may be associated with laceration of tissues which takes place during parturition.

Predisposing causes of postpartum haemorrhage can be traced to (*a*) antenatal conditions, (*b*) labour and to (*c*) delivery. Anaemia and toxaemia with their nutritional deficiencies, cardiac and pulmonary diseases all influence the stages of labour, therefore these must receive proper attention during pregnancy. Placenta praevia or toxaemia may cause antepartum haemorrhage and if this is followed by even a slight degree of bleeding after delivery the risk to the mother's life is considerable. Every precaution should be taken to avoid or correct such complications as pelvic and foetal disproportion, malpresentations, over-distension of the uterus, and other factors which cause delay and uterus atony. Relaxation as advised by Grantly Read has done much to prevent uterine atony and lacerated tissues during labour.

At the present time there are arguments about the use of sedatives in the first stage of labour, and about the risk that deep or prolonged anaesthesia may effect uterine action in the second stage. Undoubtedly the administration of nitrous oxide with air or oxygen has been a great boon in domiciliary practice. If there is delay in the second stage of labour, and forceps application is indicated, there may be difficulty or delay in obtaining the assistance of an anaesthetist. Though the administration of chloroform is now condemned by most obstetricians, there is still a place for it in emergency conditions when given by the intermittent method on an open mask; under these conditions it is the safest and easiest method. It should be avoided in all cases of toxaemia and should not be repeated. Care should be taken to avoid a solid meal within some hours of delivery.

* A paper delivered to the Bristol Medico-Chirurgical Society on 13th February, 1952.

With further development of analgesic methods such as low spinal and local infiltration, obstetric operations such as forceps and repair of lacerated tissues will become much safer.

More skill in the delivery of the infant, and the avoidance of hurried interference are more important than all the arguments for and against sedatives and anaesthetics.

In the management of labour these three points need constant attention. The patient must be given plenty of fresh air and the oxygen content of her blood must be maintained; a supply of oxygen should be at hand in every midwifery case, normal or otherwise, for it may be required if there is a haemorrhage. Exhaustion should be prevented by giving light meals, and plenty of fluids such as tea, milk or fruit drinks sweetened with glucose. Thirdly, and of much importance, the bladder should be emptied at regular intervals throughout labour, say every three hours and especially before delivery. The patient can sit on a bedpan or on a stool; but to reduce the risk of sepsis only under exceptional conditions should a catheter by employed.

If the bladder is full considerable discomfort or pain is experienced during labour and delivery. This is especially so during the third stage of labour if the uterus is compressed in order to assist delivery of the placenta. Pressure on a full bladder may be accompanied by shock. (In former times, delivery with an unemptied bladder used to be followed by vesico-vaginal fistulae, either from the foetal head or from the hand of an attendant.)

### MANAGEMENT OF THE THIRD STAGE OF LABOUR

It is most important not to interfere with the normal third stage of labour for this is one of the biggest causes of postpartum haemorrhage. In domiciliary practice once the birth of the infant has taken place the waiting relatives cannot always understand why there is delay before they can have access to the patient and the newborn baby. The attendants are therefore inclined to pay too much heed to the clock, and to shorten when possible the delay before the delivery of the placenta. What matters is the condition of the patient and not the time factor.

The British method of management of the third stage of labour hitherto has been to keep the hand on the uterus in order to prevent haemorrhage and to aid expulsion of the placenta by uterine contractions. During my attendance in the obstetric clinics of Berlin and Vienna in my early post-graduate days I was much impressed by the management of the third stage. There, after delivery, the patient was covered and allowed to rest from the fatigue of labour and delivery. At intervals the attendant placed the hand on the uterus to find out if the placenta had descended into the vagina. If not, more time was given, and no effort made to dislodge it. When it was found to have reached the vagina the patient was told to press down and expel it by means of her abdominal muscles. In this way the birth of the placenta was rarely accompanied by unusual bleeding and the third stage was not unduly prolonged. This method of management of the third stage I have taught ever since, with insistence that the patient is kept continuously under the supervision of the attendant but without interference with the progress of the third stage.

When this method is employed the patient after resting may begin to feel uterine contractions followed by some lengthening of the umbilical cord and a gush of blood. This indicates the separation of the placenta. If the attendant's hand is placed on the abdomen the uterus is felt to be high and movable with a certain amount of prominence above the symphysis pubis. The uterus if soft should be gently massaged and when it has contracted the thumb is placed outside the abdomen and the fingers behind the uterus; the uterus is then gently pressed downwards and backwards in order to expel the placenta from the vagina. As soon as the placenta appears at the vulva the hand is taken off the abdomen, and both hands cup-shaped are placed to receive the placenta and prevent sudden tearing of the membranes.

The placenta should be laid flat on a towel or dish and examined on both sides to show if it is intact. If the membranes are caught in the cervix time should be given for the uterine contraction to pass off, and then the membranes should be gently delivered by the forefingers and thumbs. The uterus can now be massaged, and the toilet of the patient completed, the binder and pad being applied at the vulva. If small portions of the placenta or pieces of membrane are missing a note to this effect should be placed on the patient's chart, but no exploration of the uterus must be made. Ergometrine 0·25 mg. to 0·5 mg., or pituitrin 5 units intramuscularly can be given, if required.

### TREATMENT OF POSTPARTUM HAEMORRHAGE

Textbooks say that blood loss over 500 cc. amounts to postpartum haemorrhage. But in practice we cannot measure the amount a patient has bled; the appearance of the sheets is unreliable and an atonic uterus may be greatly distended by blood without there being much external loss. A severe haemorrhage however soon gives rise to signs of shock. In these cases, blood transfusion may be necessary; and full blood grouping, including Rhesus factor, in pregnancy will save vital moments should a haemorrhage occur during or after delivery. All patients should therefore keep a card giving details of their blood group.

Bleeding due to lacerations is continuous and bright red in colour and may occur either during or soon after the delivery of the infant. The uterus may be contracted to some extent. Except in cases of severe shock lacerations should be repaired as soon as possible, particularly those in the region of the clitoris. If lacerations are higher, in the lateral fornices or in the lower uterine segment, the bleeding vessels should be compressed by means of pads and a binder. When the home conditions are unsuitable the patient should be sent to hospital for repair.

If the placenta is in the uterus and bleeding takes place the usual procedure is to remove the placenta as quickly as possible.

*Bimanual Removal of the Placenta.* The indication for this operation is haemorrhage which cannot be controlled by gentle massage of the uterus. It is not difficult if carried out by a skilled obstetrician in hospital, with the help of a specially qualified anaesthetist. However in the patient's home, with little assistance and indifferent anaesthesia, the operation is much more difficult especially if the patient is squirming about the bed from pain.

The patient should be on her back with legs flexed, and all precautions taken to avoid sepsis. The left hand is placed on the abdomen and controls the uterus throughout the operation. The lubricated right hand is inserted slowly into the uterus until the edge of the placenta is felt. When this has been completely separated from the uterine wall, it is grasped by the hand and gently delivered by traction on the cord. The hand remains in the uterus until inspection of the placenta has shown that no portions are left behind. This lessens the risk of sepsis by introducing the hand a second time. If the hand entering the uterus follows up the cord inside the membranes the risk of sepsis is said to be reduced; but this method makes it difficult to separate and grasp the placenta. As soon as the operation is completed the uterus can be massaged and ergometrine administered either by the intravenous or intramuscular route. Pads and binder are applied and the patient allowed to rest. A sedative and a cup of hot tea may be given if required.

*Later Bleeding.* There have been various treatments for haemorrhage after the delivery of the placenta. Hot douching is a problem in domiciliary practice. It takes time to prepare and ascertain the correct temperature, and a douching apparatus is not always carried in the bag. If too hot, a douche causes damage to the pelvic tissues with consequent sepsis. Packing the uterus with yards of sterile gauze means considerable preparation and assistance. Unless tightly packed it is inefficient in the arrest of bleeding, and anyway it prevents contraction of the uterus, is uncomfortable for the patient, and to be carried out efficiently needs an anaesthetic. This method of treatment has now largely been discarded. The most usual method now is that of bimanual compression of the uterus. One hand in the vagina pushes the anterior wall of the uterus upwards. The outer hand is placed on the abdomen and presses down the posterior wall of the uterus. This bimanual compression is carried out until no further bleeding takes place. Care must be taken to empty the bladder before applying the hands. The disadvantage of this treatment is that it takes up the whole attention of the attendant and may be prolonged, and is sometimes very tiring both for patient and attendant. When the pressure is discontinued bleeding may take place later, which again would require attention.

*The Pad and Binder.* The method I devised some years ago has given much more satisfactory results and is easy to apply. It can be used in cases of severe haemorrhage and shock, in cases of abortion and also as a temporary measure in ruptured ectopic pregnancy. The method is simple, inexpensive and available at a moment's notice. It consists of a broad many-tailed abdominal binder made of strong cotton or calico, a long pad or towel attached to the binder behind and pinned over the vulva when the binder has been applied. A smaller pad is used for pressure above the symphysis pubis to put the uterine vessels on the stretch. A sanitary towel is very useful for this purpose.

The patient is placed on her back on the spread-out binder with her legs stretched, and the flat hand above the symphysis pubis presses the uterus *upwards*, the small pad is then applied and kept in place until the binder with its overlapping tails is brought from the lower border upwards when the hand is removed. The last tails are then pinned in place. Two safety pins only are required, one for the upper last tails and one for the vulval pad in front when the binder has been applied. A safety pin can be used to keep the vulval pad attached to the posterior wall of the binder but it is more comfortable for the patient to have it already stitched. The binder can be left in position for some hours until the patient desires to pass urine.

If each midwife carried this simple outfit in her bag she need not dread post-partum haemorrhage.

